# Social Quarantine and Its Four Modes: Conceptional Exploration and the Theoretical Construction of the Policies Against COVID-19

**DOI:** 10.3389/fpubh.2021.614476

**Published:** 2021-09-23

**Authors:** Ka Lin, Ayesha Mumtaz, Mohammad Anisur Rahaman, Ka Ho Mok

**Affiliations:** ^1^College of Public Administration, Zhejiang University, Hangzhou, China; ^2^Institute of Policy Studies, Lingnan University, Hong Kong SAR, China

**Keywords:** quarantine, social quarantine, social contact, COVID-19, global health, social policy, community, pandemic

## Abstract

Building on the studies of health quarantine from a social perspective, this article explores the complex contexts of social quarantine as a mode of public health, a mode of community action and a behavioural and psychological mode of social distancing. To establish a conceptual investigation of the “social quarantine” issue, this study investigates four approaches to quarantine: public health, social administration, behavioural norms, and psychological effects. The study identifies the features of these modes and discusses their relationships. In addition, this study constructs a preventive framework for quarantine that embraces social and health policies to enrich the understanding of policy measures for social distancing and lockdown measures. On this basis, the study evaluates the strategies of policy development in response to the COVID-19 pandemic. The study concludes that these modes can reconstruct social relations and provide some basis for theoretical analysis about the features of social quarantine, which is vital for policymakers when considering national and global prevention strategies for public health.

## Introduction

The COVID-19 pandemic declared in early 2020 has stimulated academic discussions about the idea of social quarantine. With different quarantine measures adopted globally, the debate on the social effects of mandatory quarantines and widespread travel restrictions has become popular. Some regard them as doing more harm than good (1), whereas others insist they are positive measures to prevent illness. For instance, Specktor (2) commented on large-scale quarantine strategies implemented in countries such as China, Italy, and India, and their blunting the curve of COVID-19 infections in these countries, and Tognotti (3) stated that the quarantine strategy raises ethical, political, and socioeconomic problems, and that a balance is required between individual rights and the public good. These debates have impacted research conditions regarding social quarantine as a means of public health, and as an important tool used to control unexpected illnesses or to prevent epidemics.

In the global context, the World Health Organization (4) defined “quarantine” as “the restriction of activities of healthy persons who have been exposed to a communicable disease, during its period of communicability, to prevent transmission during the incubation period if infection should occur.” This quarantine ideal, as a non-pharmacological general well-being framework of policy analysis, became popular during the pandemic as a mean to curb the spread of the virus (5), especially when knowledge about the virus and its features was limited. However, the public views the need to apply social quarantine not only as a health measure, but also for social control and social administration. Brooks et al. (6) stressed that quarantine can lead to stereotypes and prejudices about certain people based on their appearance, race, culture, or national origin regardless of scientific facts; Drews (7) also regards quarantine within the parameters of human technology to provide a deeper understanding of its uses in relation to advancements made in science and medicine.

Accordingly, we need to do conceptual and theoretical work on various meanings of social quarantine and to assess its social consequences from different aspects. People apply social quarantine mechanisms to reduce social communication through two distinct strategies: mitigation and suppression (8). The former aims to reduce the infection rate through non-pharmaceutical interventions, whereas the latter takes extremely limiting measures to reduce the number of new cases. At the behavioural level, a study conducted in Italy states that when the most stringent community quarantine measures are employed, this reduces all activities in the community. It is more than evident that reduced community activity influences behavioural norms, group or household sizes, and generates feelings of social exclusion.

This study will address the variables affected by COVID-19 during social quarantine and discuss the usage and outcomes of implementing social quarantine modes. The study will adopt both macro- and micro-level analyses to interprete the ideal of social quarantine. From a macro-level perspective, the need of social quarantine should be managed to achieve a balance between the interests of public health and the emergency of economic operation. In the context of community actions and socioeconomic development, this ideal should be assessed within both normative and structural reasons, since quarantine concerns both social and economic functions. Thus, the study will evaluate the policy implications of community shutdowns and regulating people's daily lives to achieve economic reopening in response to the COVID-19 pandemic.

From a micro-level perspective, Kissler et al. (9) insisted that compulsory home quarantine and the arrangement of isolation can effectively alleviate the spread of infection, together with policy measures, including school and business closures (8, 10, 11). As argued in some policy studies, the large-scale quarantine measures at the time of the 2003 SARS epidemic were conceptualised in light of social distancing (12). Meanwhile, the quarantine issue should also be analysed at the behavioural level. Indeed, social quarantine actions must consider the psychological function of individuals and thus involved the issues of public opinion, mass reaction, and social psychology, along with their social movement and political activities.

Nevertheless, having discussed the behavioural and psychological aspects, this study mainly engages conceptual work and policy analysis on the subject rather than sociological studies of structural interpretations of the rules and behaviours of quarantine. In previous studies, some researchers showed the effect of prolonged quarantine was associated with poor mental health, particularly post-traumatic stress symptoms, anger, and avoidance behaviour (6, 13, 14), and at the behavioural level, the regulations about the behavioural rules are discussed, such as a distance of one metre between customers in bars and coffee shops and the rules reducing interpersonal interactions in social meeting. Thus, this study constructs a four-modes frame of analysis and test explanatory power of these modes in the policy practises as the strategy of prevention in the COVID-19 pandemic.

## Reviewing the Studies on Social Quarantine

Despite many studies about social quarantine measures impacting the health function of epidemic prevention, the social contexts and consequences of quarantine has not been examined thoroughly. Thus, how to evaluate the success or failure of local practises, and from what perspectives become the key issues of our analysis. The implementation of quarantine strategies should be assessed in consideration of local sociocultural contexts, not simply in the terms of health technology and caring standards. Thus, Blendon et al. (15) talked about the experience of preventing the SARS outbreak, praising mainland China, Hong Kong, Singapore, and Taiwan as successes, whereas Schabas mentioned that the mass quarantine strategy was a failure in Toronto (16). In the case of COVID-19, China again achieved remarkable results in controlling its spread by implementing strict quarantine strategies; however, some observers asserted this success could only happen in China because a similar technique was hardly used in other countries, such as the United States, India, and Pakistan (17, 18).

In the study of social consequence on a macro-level, some scholars expose the social impact of quarantine on the issue of social exclusion. Moisio (19) pointed out that social quarantine may aggravate social inequalities and class disparities since the most vulnerable groups during the pandemic were those low- and middle-income families as these groups were severely affected by market closures and months of factory lockdowns. The use of quarantine to isolate people raises the risk of social exclusion and, at the same time, often violates the freedom of outwardly healthy people, particularly people from marginalised groups who are stigmatised and discriminated against (3). In this regard, the COVID-19 crisis must have a devastating effect on vulnerable population by potential exacerbating existing unequal access to education, healthcare, and social services.

The most crucial issue in this quarantine debate is the social consequence of restricting individual mobility against individual freedom. Bensimon and Upshur (12) emphasised that the effectiveness of public health interventions should not be defined only in (absolute and objective) scientific terms but should also be conceptualised reasonably and normatively in public health decision-making. For these policy studies and evaluations, Sopory el al. (20) pointed out that often the effectiveness of quarantine is judged by some utilitarian criteria of reducing mortality and morbidity, and suggested that discussion should also include additional criteria. The protection of civil rights and protection from harm, as quarantine may impose on people without their consent. Thus, some researchers criticise the enforced use of social quarantine in the face of a dramatic health crisis because individual rights have often been trampled in the name of the public good (12, 16, 21).

Though the aforementioned studies focused on the macro-level effects of social quarantine, we should not ignore its effect at the micro-level. In previous studies, some researchers reported the harmful impacts of quarantine on mental health; for example, according to Brooks et al. (6), people under quarantine experienced negative mental health impacts, including post-traumatic stress disorder, anger, and confusion, Marjanovic et al. (22) talked about healthcare workers who were quarantined engaging in avoidance behaviours, such as minimising direct contact with patients and not reporting to work. In this regard, Tang et al. (1) reported the depression and anxiety in a group that underwent quarantine was higher than the group that did not undergo quarantine, and Caleo et al. (23) showed that the main stressors for COVID-19-infected individuals/those suspected of having the virus post-quarantine are financial issues and stigma from society. Therefore, we should consider the psychological and individual aspects of social quarantine as well.

Since the function of social quarantine can be interpreted from different angles and approaches, our analysis should look at both the macro- and micro-levels of social actions to develop the conceptual and theoretical work to respond many complicated issues to be engaged. Research shows that individuals cope better with changes in lifestyle when they plan for said changes; this has also been shown to enable better compliance with public health guidance. With regard to the policy analysis, moreover, social quarantine measures are key to discussing virus prevention and rehabilitation to solve policy decisions in addressing the conflict between both scenarios. This framework points to the importance of justifying public health intervention on the basis of its effectiveness.

## Research Design About a Four-Modes Frame of Analysis

To classify social quarantine in various contexts, we propose four basic analytic modes. First social quarantine should be referred to as a mode of public health. In the original sense, social quarantine is an exhaustive tool used to control unexpected illnesses to prevent epidemics. In Gordon's (24) terms, “quarantine” is used to contain those who are asymptomatic but not resistant to infection. This method was first used during the Black Death in the fourteenth–fifteenth centuries in the Southern and Eastern Europe (25), when a 40-day quarantine period was applied to high-risk groups. Thus, from a health perspective, social quarantine can ensure that infected individuals are distant from the general population to reduce the frequency of interpersonal communications. Hence, “quarantine” refers to the policy restricting people's movements (26), and to be effective, social quarantine as a mode of public health requires a number of health policies to prevent uncontrollable sources, hinder the transmission process, and ensure the safety of the susceptible population (27).

Social quarantine can also be a mode of community action. This makes the community service an essential element of social segregation by separating people or communities who have been exposed to an infectious disease. Therefore, community lockdowns, checkpoints, suspension of open markets, and border controls have all been used to curb the spread of COVID-19. These actions involve the interaction and collaboration of various local stakeholders, including actual and potential patients, volunteers, and local administrators. Social distancing measures to target the spread of viruses among the population may, at the community level, include the closure of schools and organisations, mandatory travel restrictions, curfews, and limitations to the sizes of parties (28). Additionally, community services maintain social distancing by isolating sick residents and keeping them under observation. A pre-existing favourable environment for health and hygiene practises makes operating or even increasing quarantine measures in a community easier for community organisations ((29–31)).

The implementation of social segregation requires the cooperation of residents, since in the quarantine actions, people participate in multifaceted and complex activities with their family, social networks, and organisations by engaging in community activities and individual behaviour (32). Thus, the adoption of community education is essential for local governments to establish an epidemic warning system to reduce people's possibly risky behaviour in social activities. In some instances, all residents of high-risk areas were encouraged to stay at home, an effective way to protect the community from exposure to infection. Thus, positive neighbourhood relationships and access to information about the disease are important for the local control of health conditions. It has also been stated that the use of quarantine to isolate people suspected of being infected—often violating the freedom of outwardly healthy people, especially people from lower classes, minorities, and marginalised groups—leads to stigmatisation and discrimination (3). Taking the time to engage community members, inform them, and to act as an effective liaison between them and district health authorities through trusted local leaders, helps implement effective segregation at a community level.

Social quarantine can also be a mode of individual behaviour. An ideal “social quarantine” demands that every member of local society should voluntarily apply social distancing. If people do not comply, the social quarantine model will not be effective. For example, wearing masks in public places should be encouraged, and elbows should be touched in greeting instead of hugging, kissing or shaking hands to reduce the frequency of interpersonal communication ((33); Smith et al., 2017). Hand washing and avoidance of crowds may be measures applied to maintain distance from interpersonal connexions. Thus, some restrictive measures should be taken to control individual behaviour, and all misconduct and improper behaviours that carry the risk of infection should be penalised to restrict people's behaviour. These requirements change the behavioural models of people's daily lives.

When the behavioural mode is implemented, social quarantine will inevitably be affected by the moral and cultural characteristics of the local society. From the perspective of “rationality,” people's behaviour is determined by social norms, the perceived benefits of quarantine, the risks of disease and the effectiveness of quarantine. Once people know about the risk of a certain disease, they formulate new rules affecting behavioural changes. Discussions on behavioural patterns must refer to the social norms and cultural background of individual behaviours. Thus, the behaviour of social quarantine will be influenced by the cultural qualities of the local society. For instance, mandatory prohibitions against praying in a church during quarantine compromised the religious lifestyle and increased the anxiety of religious individuals (34). Thus, we need to express the relationship between social isolation as a code of conduct and local morality and should formulate separate agreements according to different cultural contexts.

Lacking access to the outside world, patients may feel discomfort and a perception of risk (35), and may fear that threats may be evident (Cole, 2013). In this case, social isolation can be a mental mode comprising several sensations and feelings. Thus, a psychological mode of social quarantine also exists, as a pandemic will produce certain subjective feelings, such as danger and panic (specifically when in crowds). The vulnerability of emotional elasticity is the most evident feature of the psychological mode of social isolation. Social distancing may exacerbate loneliness and negatively affect health in the long run, as research showed that quarantine can also contribute to stress and anger (36, 37). In particular, when information and communication are insufficient, people's feelings are heightened, and they may be over sensitive to the risks to themselves or others.

Therefore, social isolation can have a negative effect on the psychological characteristics of individual behaviour. The pandemic caused a high prevalence of stigmatisation in public groups, followed by traumatic stress symptoms and insomnia, anxiety, and depressive symptoms. Thus, social segregation should be studied from a psychological perspective, together with psychiatric symptoms, mental disorders, and mental health problems, which may lead to biological and psychological abuse. As reported, quarantine measures cause up to four times as much post-traumatic stress in isolated people compared to non-isolated people (6). It can also lead to legal wrangling, messy confrontations, and poor mental health. Researchers found long quarantine periods can cause symptoms of post-traumatic stress disorder and depression in patients. People in quarantine often experience anxiety, frustration, or fear of infection, as well as negative feelings related to isolation, loneliness, anger, and perceived or actual stigma (38–41). Thus, the quarantine will greatly affect people's attitudes towards discipline, collective action, and social grouping (42).

In the case of COVID-19, the rapidly increasing numbers of disease outbreaks worldwide often caused anger and anxiety in different nations. Fear and perceived threats of socioeconomic groups may lead to intolerance and punitive attitudes towards outsiders (43, 44). The experience of fear and threat not only affects people's perception of themselves, but also influences their perception of and reaction to others—in particular, out-groups. Indeed, in the 2020s, fear during the pandemic, together with the economic difficulties associated with the increase in the prevalence of cases, drove risks usually associated with high levels of ethnocentrism. This mass psychology poses a great risk to international relations and may lead to optimism bias inducing excessive feelings of anxiety, as it influences international affairs with a hasty attitude and leads to disputes for irrational reasons.

## Policy Implications of Identifying Four Social Quarantine Modes

The four aspects of social quarantine listed above—clinical, communal, behavioural, and psychological—help us to assess the situation of anti-pandemic interventions in different contexts. This four-mode interpretation improves our understanding of the function of social quarantine and its features. We need to study the policy implication of these modes in various aspects. In defining these four modes from macro- and micro-level viewpoints. The clinical and community models refer to macro-level social approaches for epidemic control, whereas behavioural and psychological models refer to micro-level individual perspectives. From a macro-level standpoint, the COVID-19 pandemic is not only a public health crisis but also a socioeconomic emergency. This crisis led to a risk to public health, as the virus has spread globally. It poses a risk to states and may require an organised international response.

To meet the needs of social quarantine in a clinical mode, we may adopt policy measures to reduce the risk of transmitting viruses through human contact. These policy measures include smart health through long-distance diagnoses, through which doctors can advise their patients. The monitoring system, the service platform, and the home-based quarantine facilities work effectively for prevention, and moving school courses from classrooms to online platforms is also quarantine in the clinical sense. Meanwhile, a tracing system (as in China's system of green code) greatly contributes to preventing disease transmission, and the hospital grading system also helps avoid crowding of patients in hospitals, thus reducing the risk of mutual infection.

The policy measures for social quarantine in the mode of community actions include community lockdown, traffic suspension, and the ban of gathering at bars and commercial centres. Voluntary services for checkpoint monitoring in the community involves using volunteers to provide a full range of services for those under community quarantine conditions, including door-to-door delivery, control of community mobility, and decentralisation of social gatherings (45). These policy actions demonstrate the significance of “community-wide containment” to reduce infection rates at different stages of the disease (46–49). “Isolation is the separation, for the period of communicability, of known infected persons in such places and under such conditions as to prevent or limit the transmission of the infectious agent” (4).

In light of the behavioural models, social interaction is deeply rooted in human interaction and social organisation, and social quarantine refers to social distancing that reduces human interactions. In this regard, there are policies to regulate normal behaviour in a pandemic, such as keeping a distance of one metre between customers and wearing masks in shopping centres. The production of guidelines for people to reshape their behavioural model ultimately changes people's minds, thereby affecting their levels of mental health. Once social distancing becomes a norm in people's daily behaviours, they become more active in supporting the clinical mode and the community model of social quarantine, integrating the rules into their behaviour, and affecting the pace of rehabilitation.

Concerning the psychological mode, policies were created to relieve symptoms, such as trauma and anxiety in the victims of the pandemic. Loneliness and social isolation increase the burden of stress, and often have detrimental effects on psychological health, cardiovascular health, and the immune system (50). Some reports from Nigerian households in quarantine due to Middle East respiratory syndrome (MERS) show anxiety, depression, mental distress, and the influence of grief-related trauma during MERS (51, 52). Similar reports related to COVID-19 cases describe public fear, anxiety, exhaustion, and detachment from others; with feelings of uncertainty and unpredictability. Thus, policies for psychological welfare require information transparency and mass media messaging to address mass trauma and to provide comfort and entertainment for the people in quarantine at home.

Despite these four modes being identified as different, we can also perceive them as being interrelated. The technological instrument facilitates the models of clinical and community modes, which influences people's antivirus actions. The behavioural model is influenced by the rules of social quarantine imposed on human communication and affects the norms of daily life. Since quarantine practise can allow people a sense of uncertainty while limiting their movement among communities, the behaviour model influences mental health by warning of infection, which has an effect on the social quarantine of normal people. In the long run, these psychological effects are moderated with community service. Thus, the clinical need for social isolation has diminished, and community barriers have been removed through policy work on behaviour and psychological models.

Meanwhile, different societies have different policy strengths, which are also affected by their social-cultural contexts. Social distancing rules were still useful, particularly when there was no vaccine available. Alongside some common practises, such as wearing masks and changing our patterns of interpersonal connexion, we need to study the special features of their policies against the pandemic, and the four- mode framework is a practical basis for this comparative analysis. In previous studies, social quarantining has been regarded mainly as a tool to control the prevalence of a disease, and thus the clinical and public health functions are highlighted when assessing social quarantine, but social and behavioural functions are less so. Meanwhile, social quarantine has a very strong function of social administration, so there are overlapping needs of medical and social administrative functions (53). This could create some debate and cause controversy in academic and policy-making circles.

## Examples of Social Quarantine in Case Comparisons

Overall, the four-dimensional approach to social quarantine explains why returning to normal life and a normal economy is difficult due to the complicated impacts of social quarantine. Of the four modes, social quarantine must be understood in different ways. Different countries may have mutual learning practises for social quarantine strategies, and different community intervention strategies must be selected, calibrated, and implemented according to the intensity of local COVID-19 transmission to avoid the risk of spreading the virus (54). With this understanding, we can observe how different countries use social quarantine policies to respond to crises in different ways. Thus, in this section we select three cases of illustration for these modes to reveal the policy implication of these modes and their influence over the strategies on the local practises against the Covid-19. The diversity of these approaches leads us to consider the policies' contexts and different socio- political environments.

### The German Experience of the Clinical Mode

In Europe, Germany ranked first in terms of per capita gross domestic product. In this pandemic, its number of reported cases is twelve among the top 10 European economies (see Table 1), and its mortality rate is not very high (nearly 11% of infections). This performance rests on its numerous healthcare policies. In addition, people have high confidence in the developed healthcare facilities to fight the virus, as Germany is among the top five countries in the European Union that have a high number of nurses (13.2) and doctors (4.2) per 1,000 people (55). It also prepared a strong private and public laboratory sector, with nearly 200 laboratories having COVID-19 testing capacity and ventilators. More importantly, Germany's system for grading clinical diagnoses prevented patients from congregating in large hospitals (56), with only those with suspected symptoms hospitalised.

**Table 1:** Ranking of the GDP and the situation of Covid-19 in European states.

**Country/economy**	**GDP (PPP)**		**COVID-19**					
	**Share in 2019 % Europe (Eur.)**	**Rank**	**Cases**		**Deaths**		**Population Per 100,000**	
			**Total**	**Rank**	**Total**	**Rank**	**Cases**	**Deaths**
Germany	17.4	1	3,722,782	12	90,472	11	4,476	109
United Kingdom	12.3	2	4,640,511	7	127,981	7	6,836	189
France	12.2	3	5,650,315	4	109,879	9	8,688	169
Italy	8.94	4	4,253,460	9	127,291	8	7,132	213
Russia	7.36	5	5,350,919	6	130,347	6	3,667	89
Spain	6.28	6	3,764,651	11	80,689	14	7,954	170
Netherlands	4.06	7	1,679,542	20	17,727	30	9,648	102
Turkey	3.34	8	5,375,593	5	49,236	19	6,374	58
Switzerland	3.22	9	698,872	38	10,270	43	8,075	119
Poland	2.54	10	2,879,030	14	74,858	15	7,585	197
Sweden	2.38	11	1,084,636	26	14,574	35	10,502	141
Belgium	2.33	12	1,079,640	28	25,141	25	9,370	218
Austria	2.01	13	645,609	39	10,419	42	7,253	117
Norway	1.88	14	129,545	93	790	116	2,413	15
Ireland	1.73	15	269,321	68	4,941	63	5,425	100
Denmark	1.56	16	291,801	63	2,531	83	5,011	43
Finland	1.21	17	94,379	102	967	108	1,708	18
Czech Republic	1.11	18	1,666,192	21	30,283	22	15,581	283
Romania	1.1	19	1,080,323	27	32,465	20	5,589	168
Portugal	1.06	20	865,806	30	17,068	31	8,409	166
Greece	0.962	21	418,548	49	12,565	39	3,905	117
Hungary	0.766	22	807,684	33	29,879	23	8,267	306
Ukraine	0.676	23	2,230,142	16	52,053	18	5,099	119
Slovak Republic	0.479	24	391,385	53	12,502	40	7,171	229
Luxembourg	0.312	25	70,535	110	818	114	11,266	131

*Source: the data of European economies, see International Monetary Fund World Economic Outlook (October-2019), 2020.02.24. http://statisticstimes.com/economy/european-countries-by-gdp.php; The data of the Covid-19 in the Europan region, see https://voice.baidu.com/act/newpneumonia/newpneumonia, 2021.07.19*.

Meanwhile, Germany was the first place to successfully use a smart health system to implement a strategy for social quarantine. As this smart health system operated successfully in Germany, the flow of medical services to large hospitals was reduced. This promotes the idea of social distancing by avoiding crowds in hospitals. In addition, Germany demonstrated the importance of the clinical mode of social quarantine through its advanced family doctor system. In this system, general practitioners facilitated a system of distanced health services, and the contracts signed between family doctors and the local community have been widely recognised. People can contact their family doctors for consultations, diagnoses, and treatment without needing to visit hospitals.

### The Chinese Experience of Community Mode

A clinical mode of social quarantine was used in the initial phase in China by building two new hospitals (Huoshenshan and Leishenshan in Wuhan) and 14 temporary healthcare centres to manage the crisis. These actions provided quarantine facilities for people suspected of having the virus. However, the community model of social quarantine played a key role in disease prevention, thereby suppressing the local transmission rates successfully (57, 58). From the SARS experience, China recognised the value of community control as the most effective way to combat a pandemic. The most fundamental duties of the community mode are lockdowns, conducting checkpoints, community blockades, and “family outdoor restriction” policies. City lockdown policies and traffic bans or restrictions were enforced for social quarantine when the features of COVID-19 were unknown and the vaccine was in its experimental phase.

For policies to promote social quarantine through community actions, the system was supported by voluntary groups who provided services for infected (or suspected) patients isolated at their homes in the local community. Volunteers delivered door-to-door services to families in lockdown and collected their waste. Many governmental and non-governmental organisations encouraged their employees to “voluntarily” protect and monitor lockdowns to prevent the virus. With regard to psychological effects, the psychological model of social quarantine is equally applicable. As Shen et al. (59) described, contact tracing and isolation of close contacts (preventing infection before symptoms occur) can release the psychological stress of COVID-19. However, as the special feature of antivirtue actions, China has an advantage in organising a community system, which provides social services and local control through national recommendations and information guidelines.

### The Scandinavian Experience of Behavioural Mode

In Scandinavian countries, the herd immunity theory is a hot topic for popular debates, making many people resist community control to defend their right to freedom. Under the influence of herd immunity, the community mode of social quarantine is weakly implemented. People make voluntary choices between the coercive and voluntary practise of social distancing. In Sweden, people maintained daily maintenance of shops, schools, and their social lives, and applied limited virus testing in the early phase, reserving testing only for those with severe symptoms. To reserve health resources, community control is not strictly adhered to. Denmark and Norway imposed relatively strong restrictions against public gatherings, but specific decision are still dependant on the circumstances. Finland closed high schools and universities, but kindergartens and primary schools remained open from grades 1–3 to support working parents (60).

Thus, a behaviour model is stressed and residents were required to restrict unnecessary travel. However, a solution of voluntary distancing is very much subject to the behavioural norms, though there were rules restricting gatherings of more than 10 people in all these Scandinavian countries. Despite an open view on community control and lockdown policies in the clinic and community models, outreach initiatives and collaboration on communication strategies are vital to ensure compliance. Compliance with quarantine measures depends on a number of factors or strategies used by the authorities responsible for the emergent management. This leads to diverse policy practises from radical Sweden (which adopted an open-door policy) to conservative Finland, whereas Denmark and Norway were somewhere in between.

In consequence, if we calculate the number of infected cases and the mortality rate per 100,000 of the population, they were 5,011 and 43 in Denmark, respectively; 1,708 and 18 in Finland, respectively; and 2,413 and 15 in Norway, respectively. These figures indicate a very low level of infection among the Scandinavian states, but a high rate of Sweden (10,502 and 141, respectively) (see Table 1). Since success or failure of pandemic control in Finland and Sweden is highly due to their different attitude towards quarantine in their behavioural modes, the behavioural mode contributes great to explain the diversity of the outcome in the prevention of the pandemic.

Within this four-mode frame of social quarantine, we review theoretical studies and discuss different strategies of anti-virus policies. The two factors are timing, which affects the features of the development process of the pandemic in the country (and the world), and context, the sociocultural conditions and social environment in which the policy is enforced. For timing, a country may conduct a strict control policy in the clinical and community modes once the infection rate becomes high or may alternatively put increasing emphasis on the behavioural and psychological modes of social quarantine when the spread of the virus is reduced, giving authorities time to find decisive treatments, such as vaccines. In countries, such as China, Italy, and India, large-scale quarantine strategies were implemented to level their COVID-19 infection curves, but the effective of the pandemic control is varied in these societies due to their dissimilar sociocultural contexts and political institution affecting their policy practises in these four modes.

## The Relationship Between Modes

Social quarantine has been applied to reduce mortality and morbidity, and social distancing is the fundamental way to avoid transmission of infection. However, debates over social quarantine measures were engaged in by many observers, since they generate problems in maintaining people's daily lives and restoring the economic system. This study discusses the concept of social quarantine and recognises its different modes. It presents four modes of social quarantine and discusses their features, outlining them from different perspectives. Using this analysis framework, we define the complex meanings of this concept and explore the relationships of these four modes.

The effect of using the four-modes concept can be tested by the following key issues: (1) public health and the individual's daily life, (2) clinical needs and economical operation, and (3) community actions and psychological reaction. The answers will be complicated, reflected from both macro- and micro-level perspectives to integrate the conflicting needs of control and freedom. In relation to the first issue, we can expose the way to bridge the social perspective (of public health) to the individual perspective (of everyday life). With the four-mode concept, we discuss the clinical and community modes to underscore the meaning of social control, whereas the application of behavioural control and psychological modes brings the values of public interest and individual freedom together. Indeed, we can hardly capture these different states of quarantine by addressing social quarantine simply as a generalised concept without classification.

The most fundamental debate over the quarantine policy is the conflict between health and economic benefits, i.e., the aforementioned second issue. To achieve the successful control of public health against viral infection, we need to run the clinical and community modes of social quarantine with strict restrictions to mobility. This restriction will, however, create difficulties for the economy to operate. Thus, finding a way to comply with both sides of this demand is essential. The four-mode concepts may help to meet this need. For instance, we can apply the behavioural mode to continue constraining mobility across regions for public health after the end of community lockdowns. As observed, though many cities changed their policy from a lockdown to an open-door policy for economic recovery, the need (for quarantine) remains.

Thus, with the help of this four-mode model, we can apply the behavioural mode to everyday life to exercise quarantine norms. This mode embodies the principle of social quarantine into daily activities, such as handwashing, avoiding shared materials, and ventilating rooms. We can accept these behavioural norms, patterns, and regulations as the soft measures of social quarantine to achieve the desired effect instead of adopting hard measures of community lockdown and interruption to the economy. Accordingly, this four-mode classification may enable us to develop the analysis in a detailed and operational way and thus ensure the behavioural mode plays its role in social distancing.

In response to the third issue, the community mode takes a macro-level stance, whereas the psychological mode outlines individual standards of quarantine. Among these four modes, we regard the community mode as the most essential part of the quarantine concept. This mode emphasises solidarity and altruism as the normative basis of community engagement, with outreach initiatives and collaboration on communication strategies to ensure compliance. These community actions adhere to quarantine measures to flatten the infection curve, and they engage volunteers as actors in social quarantine by working as data accumulators and local inspectors at checkpoints and servicing locally isolated families. These volunteers remain in the neighbourhood to ensure that inhabitants wear a mask, test their temperature when in public, or ensure that contaminated individuals go to a quarantine centre.

Meanwhile, the psychological issue should not be neglected. The experience of quarantine may lead to long-term mental health issues; for example, a study conducted by Liu et al. (13) reported that social quarantine is understood in the sense of clinical and community work. The four-mode concept stresses the behavioural and psychological modes, which can mutually support the macro- and micro-level viewpoints or subjective and objective indicators. Some research showed that people under quarantine might express fixation on the disease and feelings of loneliness, anxiety, and depression. Loneliness may act as a stressor that produces negative effects, such as a high level of perceived stress. People also suffer from uncertainty, and may be eager to express concerns over time and seek guidelines for future development. Thus, we shall underscore the community and behavioural modes for this study, which give fundamental reasons for explaining cross-country differences.

Overall, in studies of the issue of social quarantining, researchers refer to clinical measures and community actions, which also affect the policy-making process. With this view, the tasks of community control and individual freedom, as well as economic operation, seem to be conflicting. This contrast between behavioural restrictions and individual freedom also causes criticism, as Tognotti (3) maintained that the contradiction between individual freedom and public health often causes a debate for both theoretical and practical reasons. This study analyses the conceptual and theoretical perceptions, which opens a new way of discussion on this issue by extending four dimensions of understanding about the social quarantine ideal. It not only brings out a framework of analysis for the detailed description of quarantine practises in different societies, but also provides the inner logic of these different dimensions, which helps in outlining our policy choices and development strategies to cope with the current challenge of COVID-19.

## Conclusions

As the pandemic persists, we must face conflicting tasks of disease prevention, normalisation of our lives, and the operation of the economic system. Thus, we should adopt multiple dimensions of work, perhaps not by making policy choices but rather by adopting different kinds of policies simultaneously. This demands a better understanding of the nature and features of these policy measures, with some on healthcare and illness prevention and others stimulating economic activities or normalising daily life. The proposal of adopting four modes of social quarantine helps to achieve such aims with different policies. We may use the clinical and community modes for mutual support with strict measurements to control the pandemic adopted at the same time as supporting hard control for the behavioural and psychological modes. Alternatively, once the situation is modified, we may maintain social control by implementing the behavioural and psychological modes of social distancing as the soft measures, with a lesser degree of control with regard to the clinical and community modes. This provides a large space for policy choice, and the overall effects of these policies should be carefully evaluated.

Meanwhile, we also observe the policy choices each country makes to deal with their sociocultural contexts. Taking the example of community lockdown strategy used in many European cities, this policy seems difficult to implement, as people still move around cities even after implementing this policy. This condition is in part dependant on the local tradition of community administration, as in China, where there is a mature system of community administration, which is empowered and contributes greatly to local control in this pandemic. However, this civil administration system is lacking in Scandinavian countries, so the community mode of social quarantine could hardly be implemented effectively. Thus, the application of these different quarantine modes is subject to the local conditions and “historical moments” of the pandemic, which leaves a wide space for policy choices, contextual studies, and interpretation of the policy options for development within certain sociocultural contexts. Accordingly, the presented proposal of the four-mode ideal is useful to deepen our understanding of the nature of quarantine measures in various types and the contextual and institutional reasons that limit flexibility for policy choices.

## Data Availability Statement

The raw data supporting the conclusions of this article will be made available by the authors, without undue reservation.

## Author Contributions

KL contributed to the main conceptual ideas, design and research framework, and involved in the drafting process including full manuscript revision. AM drafted the section 3 and 4, aided in interpreting the references and results. MAR contributed in drafting section 1 and 2. KM contributed to the manuscript drafting and proof reading. All authors contributed to the final revision of the manuscript.

## Funding

This research was supported by the research funds from several resources, including the National Social Science Fund of China (19ASH016) and the Chinese post doc Foundation.

## Conflict of Interest

The authors declare that the research was conducted in the absence of any commercial or financial relationships that could be construed as a potential conflict of interest.

## Publisher's Note

All claims expressed in this article are solely those of the authors and do not necessarily represent those of their affiliated organizations, or those of the publisher, the editors and the reviewers. Any product that may be evaluated in this article, or claim that may be made by its manufacturer, is not guaranteed or endorsed by the publisher.
