# Corrigendum: Social Quarantine and Its Four Modes: Conceptional Exploration and the Theoretical Construction of the Policies Against COVID-19

**DOI:** 10.3389/fpubh.2021.793721

**Published:** 2021-11-25

**Authors:** Ka Lin, Ayesha Mumtaz, Mohammad Anisur Rahaman, Ka Ho Mok

**Affiliations:** ^1^College of Public Administration, Zhejiang University, Hangzhou, China; ^2^Institute of Policy Studies, Lingnan University, Hong Kong SAR, China

**Keywords:** quarantine, social quarantine, social contact, COVID-19, global health, social policy, community, pandemic

In the published article, there was an error in Title. Instead of “Polices,” it should be “Policies.”

In the original article, there was a formatting mistake in Table 1 as published. The corrected Table 1 appears below.

**Table 1:** Ranking of the GDP and the situation of Covid-19 in European states.

**Country/economy**	**GDP (PPP)**		**COVID-19**					
	**Share in 2019 % Europe (Eur.)**	**Rank**	**Cases**		**Deaths**		**Population Per 100,000**	
			**Total**	**Rank**	**Total**	**Rank**	**Cases**	**Deaths**
Germany	17.4	1	3,722,782	12	90,472	11	4,476	109
United Kingdom	12.3	2	4,640,511	7	127,981	7	6,836	189
France	12.2	3	5,650,315	4	109,879	9	8,688	169
Italy	8.94	4	4,253,460	9	127,291	8	7,132	213
Russia	7.36	5	5,350,919	6	130,347	6	3,667	89
Spain	6.28	6	3,764,651	11	80,689	14	7,954	170
Netherlands	4.06	7	1,679,542	20	17,727	30	9,648	102
Turkey	3.34	8	5,375,593	5	49,236	19	6,374	58
Switzerland	3.22	9	698,872	38	10,270	43	8,075	119
Poland	2.54	10	2,879,030	14	74,858	15	7,585	197
Sweden	2.38	11	1,084,636	26	14,574	35	10,502	141
Belgium	2.33	12	1,079,640	28	25,141	25	9,370	218
Austria	2.01	13	645,609	39	10,419	42	7,253	117
Norway	1.88	14	129,545	93	790	116	2,413	15
Ireland	1.73	15	269,321	68	4,941	63	5,425	100
Denmark	1.56	16	291,801	63	2,531	83	5,011	43
Finland	1.21	17	94,379	102	967	108	1,708	18
Czech Republic	1.11	18	1,666,192	21	30,283	22	15,581	283
Romania	1.1	19	1,080,323	27	32,465	20	5,589	168
Portugal	1.06	20	865,806	30	17,068	31	8,409	166
Greece	0.962	21	418,548	49	12,565	39	3,905	117
Hungary	0.766	22	807,684	33	29,879	23	8,267	306
Ukraine	0.676	23	2,230,142	16	52,053	18	5,099	119
Slovak Republic	0.479	24	391,385	53	12,502	40	7,171	229
Luxembourg	0.312	25	70,535	110	818	114	11,266	131

In the original article, there was an error on Page 2, the word “at” was used incorrectly, and should be “as,” the correct text appears below.

“Moisio (19) pointed out that social quarantine may aggravate social inequalities and class disparities since the most vulnerable groups during the pandemic were those low- and middle-income families as these groups were severely affected by market closures and months of factory lockdowns.”

In the original article, there was an error on Page 3, the word “theological” was used incorrectly, and should be written as “theoretical,” the correct text appears below.

“Since the function of social quarantine can be interpreted from different angles and approaches, our analysis should look at both the macro- and micro-levels of social actions to develop the conceptual and theoretical work to respond many complicated issues to be engaged.”

In the original article, there was an error on Page 5, the word “seventh” was used incorrectly, and should be written as “twelve,” the correct text appears below.

“In Europe, Germany ranked first in terms of per capita gross domestic product. In this pandemic, its number of reported cases is twelve among the top 10 European economies (see Table 1), and its mortality rate is not very high (nearly 11% of infections).”

The authors apologize for this error and state that this does not change the scientific conclusions of the article in any way. The original article has been updated.

## Publisher's Note

All claims expressed in this article are solely those of the authors and do not necessarily represent those of their affiliated organizations, or those of the publisher, the editors and the reviewers. Any product that may be evaluated in this article, or claim that may be made by its manufacturer, is not guaranteed or endorsed by the publisher.

